# Effect of the p38 MAPK inhibitor doramapimod on the systemic inflammatory response to intravenous lipopolysaccharide in horses

**DOI:** 10.1111/jvim.15847

**Published:** 2020-07-23

**Authors:** Jennifer Bauquier, Elizabeth Tudor, Simon Bailey

**Affiliations:** ^1^ Department of Veterinary Clinical Sciences, Melbourne Veterinary School, Faculty of Veterinary and Agricultural Sciences University of Melbourne Werribee Victoria Australia; ^2^ Department of Veterinary Biosciences, Melbourne Veterinary School, Faculty of Veterinary and Agricultural Sciences University of Melbourne Parkville Australia

**Keywords:** cytokine, endotoxemia, equine, sepsis, SIRS

## Abstract

**Background:**

Doramapimod, a p38 MAPK inhibitor, is a potent anti‐inflammatory drug that decreases inflammatory cytokine production in equine whole blood in vitro. It may have benefits for treating systemic inflammation in horses.

**Objective:**

To determine whether doramapimod is well tolerated when administered IV to horses, and whether it has anti‐inflammatory effects in horses in a low‐dose endotoxemia model.

**Animals:**

Six Standardbred horses.

**Methods:**

Tolerability study, followed by a blinded, randomized, placebo‐controlled cross‐over study. Horses were given doramapimod, and clinical and clinicopathological variables were monitored for 24 hours. Horses then were treated with doramapimod or placebo, followed by a low dose infusion of lipopolysaccharide (LPS). Clinical variables (heart rate, rectal temperature, noninvasive blood pressure), leukocyte count and tumor necrosis factor alpha (TNF‐α) and interleukin‐1 beta (IL‐1β) concentrations were measured at multiple time points until 6 hours post‐LPS infusion.

**Results:**

No adverse effects or clinicopathological changes were seen in the safety study. When treated with doramapimod as compared to placebo, horses had significantly lower heart rates (*P* = .03), rectal temperatures (*P* = .03), and cytokine concentrations (*P* = .03 for TNF‐α and IL‐1β), and a significantly higher white blood cell count (*P* = .03) after LPS infusion.

**Conclusions and Clinical Importance:**

Doramapimod has clinically relevant anti‐inflammatory effects in horses, likely mediated by a decrease in leukocyte activation and decrease in the release of pro‐inflammatory cytokines. To evaluate its potential as a novel treatment for systemic inflammatory response syndrome in horses, clinical trials will be necessary to determine its efficacy in naturally occurring disease.

**Abbreviations**
DMSO: dimethyl sulfoxide
IL‐1β: interleukin‐1 beta
LPS: lipopolysaccharide
LTA: lipoteichoic acid
p38 MAPK: p38 mitogen‐activated protein kinase
PGN: peptidoglycan
SIRS: systemic inflammatory response syndrome
TACE: TNF‐α converting enzyme
TLR: toll‐like receptor
TNF‐α: tumor necrosis factor alpha

## 1 INTRODUCTION

Horses with clinical diseases such as colitis, strangulating colic, enteritis, pleuropneumonia, and retained fetal membranes are at risk of developing severe systemic inflammatory response syndrome (SIRS).^1^ Bacterial toxins such as lipopolysaccharide (LPS), lipoteichoic acid (LTA), and peptidoglycan (PGN) activate a cascade of events that lead to the release of inflammatory cytokines from leukocytes.^2^, ^3^ Consequences of this release include cardiovascular compromise, multiple organ dysfunction, laminitis, and death.^1^, ^2^, ^3^ Diseases leading to SIRS remain substantial causes of mortality in affected horses.^4^, ^5^, ^6^

A major part of the development of SIRS is activation of leukocytes and platelets by bacterial toxins, which in turn leads to the production of inflammatory cytokines, including the downstream effects of these mediators.^1^, ^2^, ^3^ Conventional treatments for SIRS in horses such as flunixin meglumine, polymyxin‐B, hyperimmune plasma, and pentoxyfylline are accompanied by adverse effects. The most effective and commonly used anti‐inflammatory drugs in this situation (nonsteroidal anti‐inflammatory drugs) work at a site much farther downstream from leukocyte activation, principally acting to decrease prostanoids. Furthermore, clinical efficacy based on randomized, placebo‐controlled clinical trials either is lacking or equivocal for many existing treatments.^7^, ^8^, ^9^, ^10^, ^11^, ^12^, ^13^, ^14^, ^15^ Therefore safer, more effective drugs for the treatment of SIRS in horses are needed.

The enzyme p38 MAPK is a key signaling molecule leading to transcription of inflammatory cytokines within equine leukocytes and platelets.^16^, ^17^ Doramapimod (BIRB 796 BS) is an aromatic, heterocyclic compound with high affinity for p38 MAPK and an inhibitory effect on its action.^18^ Doramapimod has been investigated as a potential treatment for sepsis in human patients with initially promising results,^19^, ^20^ but it failed to increase 28 day survival in phase II clinical trials.^21^ Regardless, doramapimod might have potential for the treatment of SIRS in horses, because the severe comorbidities of septic human patients generally are not present in horses with SIRS.

In previous studies, doramapimod initially was tested in vitro, where it potently inhibited tumor necrosis factor alpha (TNF‐α) and interleukin‐1 beta (IL‐1β) production when whole blood aliquots were stimulated using toxins from gram‐positive and gram‐negative bacteria.^22^, ^23^ However, before doramapimod could be tested in naturally occurring cases of sepsis (involving ≥1 different toxins), a proof‐of‐concept study was necessary in experimentally induced endotoxemia. The only established experimental model of endotoxemia in horses that also is well‐characterized and safe is the low dose LPS challenge model.^24^, ^25^

Therefore, our first aim was to establish the tolerability and safety of doramapimod in horses after IV administration, ensuring that it did not precipitate in plasma and cause any adverse effects on organ function or clinical signs. The second aim was to determine the effect of doramapimod on SIRS in response to LPS in horses as an initial step before investigation in a clinical setting. Our specific hypotheses were that doramapimod would be safe to administer IV in a specifically designed IV formulation, and that it would significantly decrease the inflammatory response to IV LPS in horses.

## 2 MATERIALS AND METHODS

The study was approved by the University of Melbourne Animal Ethics Committee. As an initial step, and before the LPS challenge portion of the study, the tolerability of doramapimod in a formulation for IV use was determined. First, it was determined that the drug remained in solution in equine plasma without precipitating, by adding it to aliquots of equine plasma ex vivo, at room temperature, at concentrations of 1 ng/mL to 10 μg/mL. Previous attempts to dissolve doramapimod in dimethyl sulfoxide (DMSO) and polyethylene glycol‐400 resulted in rapid precipitation in equine plasma and such formulations were not deemed safe for in vivo administration. Dissolution of doramapimod in (2‐hydroxypropyl) β‐cyclodextrin resulted in no precipitation and this formulation was used for IV administration to horses. It was then further tested by IV administration to 6 healthy Standardbred horses (5 geldings and 1 mare; median age, 9 years; range, 6‐16 years). Doramapimod (Selleck Chemicals, Houston, Texas) was prepared 12 to 16 hours before administration, by dissolving a dose of 0.5 mg/kg calculated for each horse in 100 mg/kg of (2‐hydroxypropyl) β‐cyclodextrin (Sigma‐Aldrich, Sydney, NSW, Australia) which had been previously dissolved in sterile 0.9% sodium chloride (250 mL final volume). This solution then was filtered sterilely using a 22 μm syringe filter in a biosafety cabinet. This preparation was kept chilled for the 12 to 16 hours it was stored until use, and no precipitation was observed. Stability data for this particular method of preparation of doramapimod is lacking, but our use of this preparation in vitro suggested it would be effective when stored under these conditions for a shortperiod of time. The doramapimod preparation was brought to room temperature and administered to the horses by slow IV injection through a jugular catheter (Angiocath, B‐D, North Ryde, NSW, Australia) over 2 minutes. Clinical variables (heart rate, respiratory rate, rectal temperature, demeanor, intestinal motility, mucous membrane color, and capillary refill time) were recorded at baseline and 15, 60, 90, 120 180, 240, 300, and 360 minutes after injection, and hematology and plasma biochemistry variables were measured at baseline and 24 hours after administration. Horses were monitored for 5 days after administration of doramapimod.

After a washout period of at least 2 weeks, the same 6 horses were used in a cross‐over design for the LPS challenge. Three horses were randomly chosen and assigned to the doramapimod group for the first phase, with the other 3 horses assigned to the placebo (control) group. For the second phase (crossover), horses were assigned to the group to which they had not been assigned in phase I. A washout period of at least 2 weeks was allowed before horses entered the second phase. Horses were deemed to be clinically healthy based on physical examination and hematology and plasma biochemistry results. Doramapimod was prepared as described above. Horses receiving placebo were given 100 mg/kg of (2‐hydroxypropyl) β‐cyclodextrin dissolved in 0.9% saline (250 mL final volume) IV which also was filtered sterilely in the same manner. On the day of each experiment, an IV catheter was placed in either the left or right jugular vein. Baseline clinical variables (heart rate, respiratory rate, rectal temperature, blood pressure measurements) were recorded and blood samples were taken before administration of doramapimod and LPS. A dose of 0.5 mL/kg body weight of the doramapimod solution (0.5 mg/kg doramapimod in 100 mg/kg cyclodextrin, as extrapolated from the human literature) was administered IV over approximately 2 minutes, followed immediately by an infusion of LPS at a dose of 30 ng/kg administered IV over 30 minutes. The beginning of the LPS infusion was recorded at time 0. Clinical variables and blood samples for cytokine analysis and white blood cell count (heparin and EDTA tubes [Vacutainer, BD, North Ryde, NSW, Australia], respectively) were taken at baseline (before doramapimod and LPS administration), and at 15, 30, 45, 60, 75, 90, 105, 120, 150, 180, 210, 240, 270, 300, 330, and 360 minutes after the beginning of the LPS infusion. Noninvasive systolic and diastolic blood pressure measurements were taken at baseline and at 120 minutes using an appropriately sized cuff placed at the base of the tail (BpTRU oscillometric blood pressure monitor; VSM MedTech Ltd, Vancouver, Canada). Measurements were taken 3 times and the mean of the 3 measurements used as the final blood pressure result for each horse. All blood samples were collected from the IV catheter and stored chilled until processed (maximum, 6 hours). One investigator (JB), responsible for collection of clinical data, leukocyte counts and cytokine assays was blinded to group assignment.

Heparinized blood samples for cytokine analysis were centrifuged at 1500 rpm for 10 minutes, and plasma stored in 3 × 1.5 mL aliquots at −80°C. Leukocyte counts were performed using a Coulter Counter (Beckman Coulter Australia, Lane Cove, NSW, Australia) with threshold set at 4 μm.

### 2.1 TNF‐α assay

Analysis of TNF‐α was conducted on stored samples using the murine fibroblast L929 bioassay, previously validated for use in equine plasma.^25^, ^26^ Briefly, L929 cells (ECACC cell lines; purchased through Sigma‐Aldrich Pty Ltd, Sydney, Australia) were cultured in Dulbecco's modified Eagle's medium (DMEM) containing 10% fetal calf serum, 100 units/mL penicillin and 0.1 mg/mL streptomycin at 37°C in 5% CO_2_. Recombinant equine TNF‐α (Thermo Scientific Inc, Rockford, Illinois; 7.8‐4000 pg/mL) was used for the standard curve. Cells were pretreated with 20 μg/mL actinomycin D to sensitize them to TNF‐dependent killing, and samples were added to duplicate wells of a 96‐well plate. Plates were incubated at 37°C in 5% CO_2_ for 24 hours and the assay then was developed using the tetrazole dye, 3‐(4,5‐dimethylthiazol‐2‐yl)‐2,5‐diphenyltetrazolium bromide (MTT reagent) for 8 hours. The assay then was developed by addition of 100 μL detergent reagent into each well, followed by incubation at 37°C for 4 hours, and plates were read at an absorbance of 560 nm with a reference wavelength of 690 nm (Synergy H1 Hybrid Microplate Reader, BioTek, Winooski, Vermont). The TNF‐α concentrations were determined from the standard curve on each plate, using Gen5 Microplate Reader Software (BioTek, Winooski, Vermont).

### 2.2 IL‐1β assay

Interleukin‐1β was measured using a human melanocyte A375 bioassay (Cells purchased from ATCC, Manassas, Virginia) previously validated for use with equine plasma.^26^ The A375 cell line is very sensitive to killing by IL‐1β. Cell culture technique was the same as that used for the L929 cells. For the assay, standard was prepared using recombinant equine IL‐1β (Kingfisher Biotech, Saint Paul, Minnesota) from 0 to 200 ng/mL. Standard curve and samples all were plated in duplicate, with a separate standard curve plated on each 96‐well plate. Processing of the assay and reading of the plates then was performed as for the L929 bioassay, with the exception of a lower concentration (10 μg/mL) of actinomycin D being added, and a longer incubation time (96 hours) being required before development of the assay.

### 2.3 Statistical analysis

Data was tested for normality using a Shapiro‐Wilk test. Most data was normally distributed, but where not normally distributed (TNF‐α and IL‐1β bioassays), data was log transformed before statistical analysis. The peak value and area under the curve (calculated from baseline) was determined for heart rate, respiratory rate, rectal temperature, TNF‐α concentration and IL‐1β concentration for each group and compared using a paired *t* test. In addition to peak values, area under the curve (AUC) was analyzed to allow comparison of measurements over the complete time course. As only 2 measurements were taken for systolic and diastolic blood pressures (baseline and 120‐minute time points), and the change between these 2 time points was analyzed to compare the increases observed in the control vs treatment groups using a paired *t* test.

White blood cell count was analyzed as change from baseline, because of the greater importance of change in white blood cell count rather than absolute number. However, the lowest absolute white cell count (nadir) for each horse also was analyzed in order to remain consistent with analysis of other variables. The maximum decrease in white blood cell count from baseline was analyzed using a paired *t* test. The lowest white blood cell count for each horse in each group also was analyzed using a paired *t* test.

For all statistical analyses a *P* value of <.05 was considered significant.

## 3 RESULTS

Doramapimod is hydrophobic, and when it was dissolved in DMSO (undiluted or 50% aqueous solution) and then added to equine plasma, it immediately precipitated. Therefore, it was clear such a formulation would not be suitable for IV use. However, dissolving it in (2‐hydroxypropyl) β‐cyclodextrin (a ring‐shaped compound with hydrophobic core and hydrophilic exterior) allowed doramapimod to remain in solution in equine plasma. When doramapimod was given IV in (2‐hydroxypropyl) β‐cyclodextrin to healthy horses, it was well tolerated and no adverse effects were observed. All clinical variables remained within normal limits, and hematology and plasma biochemistry results obtained 24 hours after the initial dose were within reference ranges in all horses (data not shown). No clinical abnormalities were detected in the 5 days after doramapimod administration.

**FIGURE 1:**
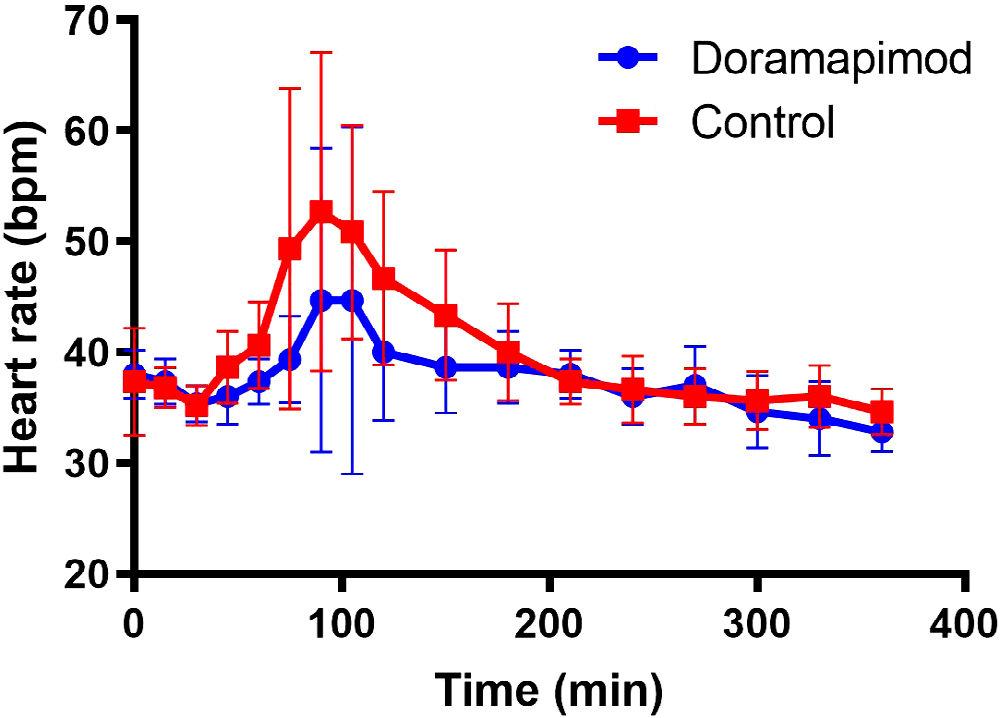
Effect of doramapimod on the response of heart rate to lipopolysaccharide (LPS) infusion. Symbols represent the mean and bars represent SD. Heart rate did not increase as much in the doramapimod group compared to the control group in response to LPS infusion. Both peak heart rate and area under the curve for heart rate were significantly lower in the doramapimod group compared to the control group (*P* = .02 and *P* = .04, respectively)

When the LPS challenge was performed, all horses showed clinical signs of muscle fasciculations, tachycardia, and increased rectal temperature transiently after LPS infusion, which are typical clinical signs in this model. However, these changes were notably less pronounced in the doramapimod group. Differences in heart rate at each time point are shown in Figure 1. Peak heart rate was significantly lower in the doramapimod group (mean ± SD = 47 ± 14.5 bpm) compared to controls (mean ± SD = 57 ± 11.2 bpm; *P* = .02). Area under the curve for heart rate was significantly lower for the doramapimod group (mean ± SD = 1050 ± 824.0) compared to controls (mean ± SD = 1862 ± 792.2; *P* = .04).

**FIGURE 2:**
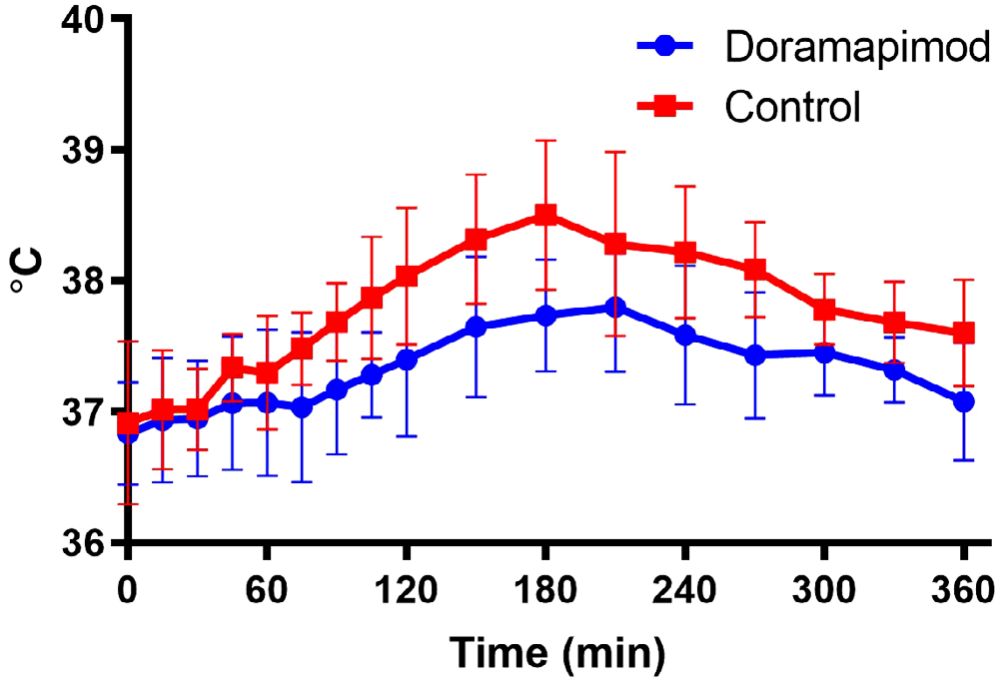
Effect of doramapimod on the response of rectal temperature to lipopolysaccharide (LPS) infusion. Symbols represent the mean and bars represent SD. Rectal temperature did not increase as much in the doramapimod group compared to the control group in response to LPS infusion. Peak rectal temperature was not significantly different between the doramapimod and control groups (*P* = .15); however, area under the curve for rectal temperature was significantly lower in the doramapimod group compared to controls (*P* = .02)

Differences in rectal temperature between groups at each time point are shown in Figure 2. Peak rectal temperature was not significantly different between groups (doramapimod mean ± SD = 38.1 ± 0.39°C, control mean ± SD = 38.4 ± 0.58°C; *P* = .15); however, AUC for rectal temperature was significantly lower in the doramapimod group (mean ± SD = 175.6 ± 105.1) compared to controls (mean ± SD = 344.2 ± 165.3; *P* = .02).

**FIGURE 3:**
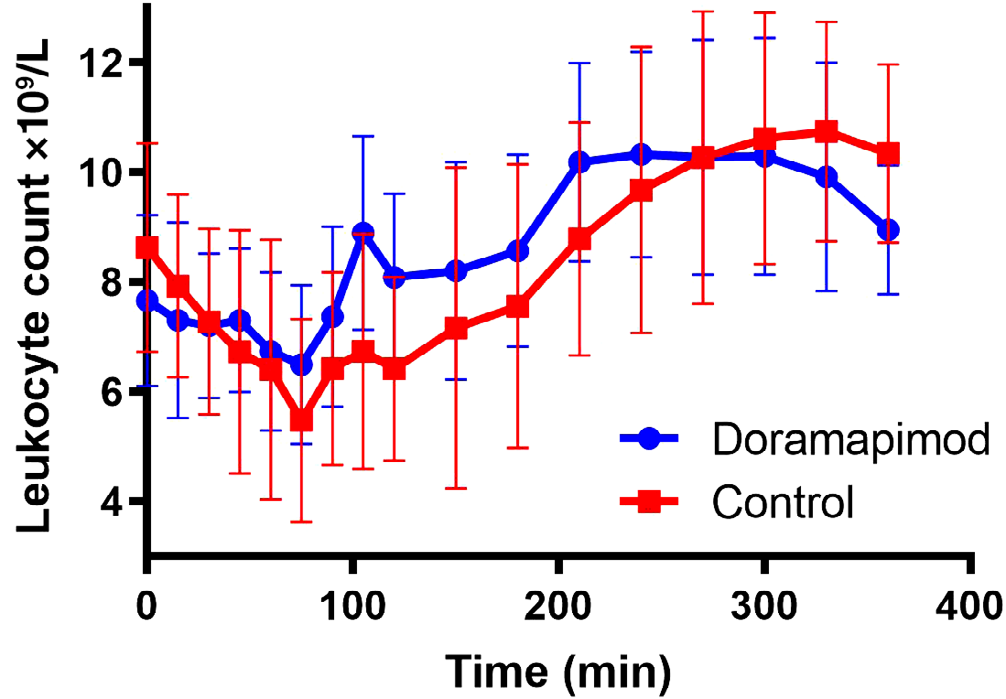
Overall effect of doramapimod on leukocyte count (WCC). Symbols represent the mean and bars represent SD. The doramapimod group had a significantly lessened decrease in leukocyte count caused by lipopolysaccharide infusion compared to controls (*P* = .003). The lowest recorded leukocyte count obtained was significantly higher in the doramapimod group compared to the control group (*P* = .003)

Systolic blood pressure increased significantly from baseline to 120 minutes in the control group (systolic increase of 17.3 ± 7.8 mm Hg [mean ± SD]) compared with the doramapimod group (systolic increase of 4.3 ± 7.5 mm Hg [mean ± SD]; *P* = .02). The increase in diastolic blood pressure from baseline to 120 minutes was not significantly different between the control group (11 ± 4.7 mm Hg [mean ± SD] and the doramapimod group (6.2 ± 7.3 mm Hg [mean ± SD]; *P* = .11). Differences in leukocyte count between groups at each time point are shown in Figure 3. The lowest leukocyte count also was significantly lower in the control group (mean ± SD = 5.236 ± 1.662 × 10^9^/L) compared to the doramapimod group (mean ± SD = 6.363 ± 1.467 × 10^9^/L; *P* = .003). The decrease in leukocyte count from baseline was significantly greater in the control group (mean ± SD = 3.39 ± 1.632 × 10^9^/L) compared to the doramapimod group (mean ± SD = 1.296 ± 0.882 × 10^9^/L; *P* = .003). Doramapimod therefore decreased the severity of leukopenia caused by LPS.

**FIGURE 4:**
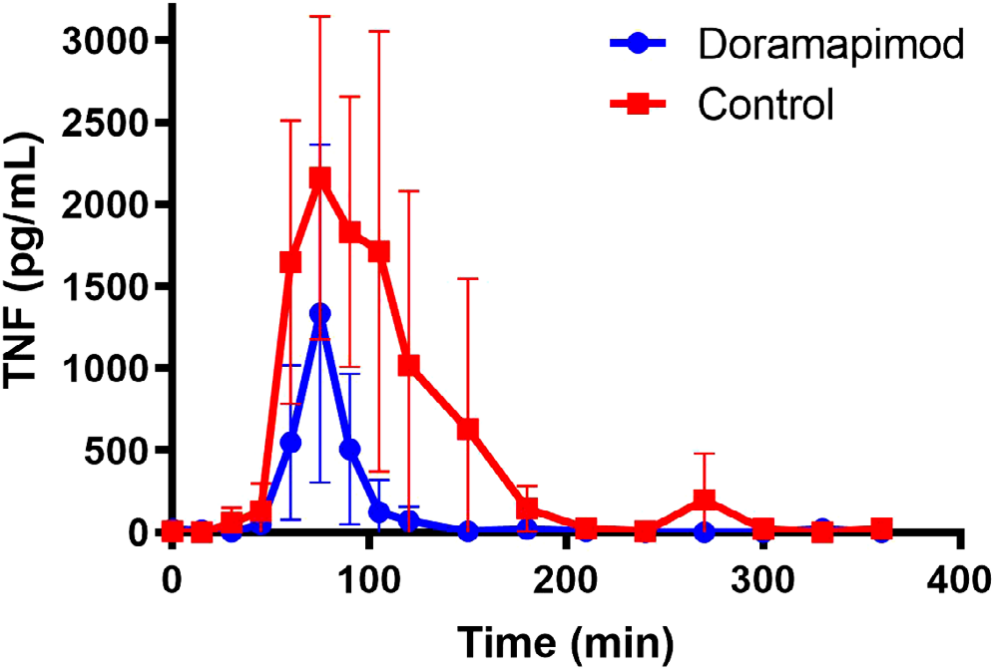
Effect of doramapimod on tumor necrosis factor alpha (TNF‐α) concentration. Symbols represent the mean and bars represent SD. TNF‐α did not increase as much in the doramapimod group compared to the control group in response to lipopolysaccharide infusion. Area under the curve was significantly greater in the control group compared to doramapimod group (*P* = .02); however, peak TNF‐α concentration was not significantly different between groups (*P* = .12)

Differences in TNF‐α between groups at each time point are shown in Figure 4. The peak TNF‐α concentration was not significantly lower in the doramapimod group (median [interquartile range, IQR] 1747 [222‐2220] pg/mL) compared to controls (median [IQR], 2800 [1435‐3432] pg/mL; *P* = .12). When comparing AUC for TNF‐α production, AUC was significantly decreased in the doramapimod group (median [IQR], 57 925 [7748‐63 709]) compared to controls (median [IQR] 170 808 [81636‐272 194]; *P* = .02).

**FIGURE 5:**
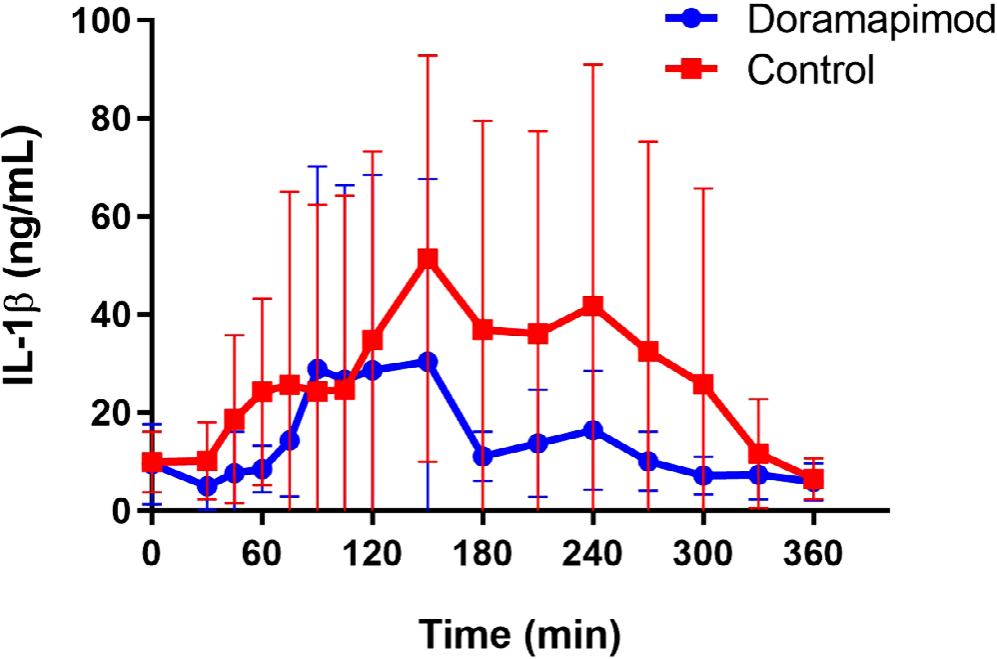
Effect of doramapimod on interleukin‐1 beta (IL‐1β) concentration. Symbols represent the mean and bars represent SD. The peak IL‐1β concentration was lower in the doramapimod group compared to the control group which approached significance (*P* = .08). Area under the curve for IL‐1β production was significantly lower in the doramapimod group compared to the control group (*P* = .02)

Differences in IL‐1β between groups at each time point are shown in Figure 5. The peak IL‐1β concentration was numerically lower in the doramapimod group (median [IQR], 23.67 [13.76‐62.94] ng/mL) compared to the control group (median (IQR), 45.62 (24.17‐105.0) ng/mL), but not significantly (*P* = .08). When comparing AUC for IL‐1β production, AUC was significantly lower in the doramapimod group (median [IQR] 2284 [756‐5795]) compared to controls (median [IQR] 3277 [1685‐16 897]; *P* = .02).

## 4 DISCUSSION

Doramapimod significantly decreased the systemic inflammatory effects of LPS administered IV in horses. By inhibiting p38 MAPK, doramapimod decreases transcription and therefore production of TNF‐α and IL‐1β, 2 major inflammatory cytokines in SIRS.^2^ When administered to animals, LPS increases heart rate, rectal temperature, blood pressure and production of TNF‐α and decreases leukocyte count because of its systemic pro‐inflammatory effects.^27^, ^28^, ^29^ By inhibiting p38 MAPK using doramapimod in horses in vivo, the substantial decrease in the LPS‐induced effects is encouraging. In in vitro studies in horses, p38 MAPK inhibition in platelets has been shown to occur using other molecules.^16^, ^30^ Similarly, studies in animal models of human disease indicate a decrease in inflammatory cytokine production when p38 MAPK inhibitory molecules are administered.^31^, ^32^, ^33^ As found in our study, healthy human subjects given doramapimod before LPS administration had significantly decreased clinical signs of endotoxemia, including production of inflammatory cytokines, neutrophil activation and C‐reactive protein release.^20^

The enzyme p38 MAPK comprises 4 different isotypes: α and β which are ubiquitous, γ which is found mainly in skeletal muscle, and δ which is found mainly in pancreas, small intestine and testicular tissue.^34^ Doramapimod inhibits p38α, β and γ isotypes,^35^ but its main anti‐inflammatory effects likely arise from p38α inhibition.^36^ Activated p38 MAPK exerts its effects either directly by phosphorylating transcription factors or indirectly by activating downstream kinases, which then phosphorylate transcription factors for inflammatory cytokines.^34^, ^37^ The enzyme p38 MAPK also has been shown to have post‐transcriptional effects, most notably stabilization of mRNA encoding common inflammatory mediators.^38^, ^39^, ^40^ Because inhibition of p38 MAPK has resulted in decreased cytokine production in several studies, it has drawn interest as a target for anti‐inflammatory treatment.^19^, ^20^, ^30^, ^36^, ^41^, ^42^, ^43^, ^44^

Our results showed that doramapimod inhibited production of both TNF‐α and IL‐1β. In both the control and doramapimod groups, plasma TNF‐α concentrations increased quickly and peaked at 75 minutes before decreasing quickly again, because TNF‐α mRNA is rapidly degraded after the inciting stimulus has passed.^45^ Tumor necrosis factor‐α usually is the first cytokine detected after LPS administration in vivo in humans and in animal models.^46^, ^47^ This is partly because pro‐TNF‐α exists as a transmembrane molecule, with the active component on the extracellular surface and the remaining portion of the peptide on the intracellular side, allowing more rapid release of TNF‐α into the circulation. Doramapimod significantly decreased release of TNF‐a into the circulation. The peak in IL‐1β occurred between 135 and 150 minutes. Once pro‐IL‐1β is cleaved to active IL‐1β, a further step of cell secretion still is required.^48^ Because doramapimod works by decreasing cytokine transcription through p38 MAPK inhibition, the suppression of the IL‐1β response caused by doramapimod may be related to inhibition of IL‐1β transcription induced either by LPS or the initial wave of TNF‐α release. However, a post‐transcriptional effect in translation of IL‐1β also is possible because of the role p38 MAPK also plays at this level.^38^, ^39^, ^40^

Doramapimod was not pursued as a treatment for sepsis in humans because of a lack of improvement in 28‐day survival and complications of mild hepatotoxicity.^21^, ^49^ Because the majority of horses that develop SIRS typically do not have comorbidities such as neoplasia, type 2 diabetes and smoking‐related illnesses that commonly affect prognosis in human sepsis patients,^50^ the effect on survival might be more favorable in horses. Furthermore, hepatotoxicity appears to occur only with prolonged administration of doramapimod in humans,^36^, ^49^, ^51^, ^52^ which is not likely to be problematic in horses with SIRS simply because the disease course tends to result in recovery, euthanasia or death of the horse within a relatively short period of time. Naturally occurring sepsis, however, is an extremely complex disease process, and lack of effective drugs for its treatment likely reflects inability of most single compounds to effectively dampen the complicated and incompletely understood cell signaling mechanisms that occur. Inflammation after IV LPS administration is not as complex, nor does it follow the same time course, as does naturally occurring sepsis. It does however activate numerous systemic inflammatory pathways and is a useful model for proof‐of‐concept studies such as ours.

Despite discontinuation of doramapimod in sepsis trials in humans, studies of doramapimod and related compounds have continued in clinical trials for human patients with chronic immune‐mediated inflammatory diseases such as rheumatoid arthritis and Crohn's disease.^36^, ^44^ However, similar problems of lack of efficacy and increases in liver enzyme activity with prolonged administration have been found, and several investigators have concluded that it is unlikely that inhibition of p38 MAPK alone is sufficient for successful treatment.^36^, ^51^, ^52^, ^53^ One interesting finding of some of these studies is that decreased concentrations of inflammatory markers such as C‐reactive protein are transient and return to baseline after 1 week.^44^, ^51^ As mentioned above, the critical period for SIRS in horses is likely still to be within this time frame. Therefore, efficacy might be expected to be higher in short‐term inflammatory diseases such as SIRS in horses, rather than in chronic inflammatory diseases, or prolonged diseases associated with sepsis in humans. Furthermore, doramapimod might be beneficial as an adjunctive treatment with nonsteroidal anti‐inflammatory drugs and other anti‐inflammatory treatments in horses with SIRS, as opposed to being a solitary anti‐inflammatory treatment.

Several studies have investigated other compounds using low‐dose LPS models in horses^7^, ^10^, ^24^, ^54^, ^55^, ^56^ but comparisons among studies are difficult because of differences in LPS dose used, route of administration, cytokine analysis techniques and timing of treatment in relation to LPS infusion. Therefore, it is difficult to compare the efficacy of doramapimod to other studies of anti‐inflammatory drugs using LPS challenge models in horses.

The dose of doramapimod used was extrapolated from the human medical literature,^19^, ^20^ but was given IV in our study as opposed to PO in studies of humans. This decision was made because the PO bioavailability of doramapimod in the horse currently is unknown, and also because many horses with SIRS have gastrointestinal disease, and therefore abnormal gastrointestinal function, making the IV route a better option. Ideally, the pharmacokinetics and pharmacodynamics of doramapimod in horses would have been fully characterized before the study, but ours was a proof‐of‐concept study and, based on the in vitro dose response curve data and blood volume of the horse, it was calculated that this dose would be sufficient to produce a physiologically relevant and measurable effect. A higher dose could have been more effective, but cost considerations and logistical factors precluded use of higher doses.

Our study was designed to demonstrate proof‐of‐concept, and therefore the experimental model used differs from a clinical scenario. Administration of doramapimod before LPS infusion does not replicate a clinical scenario, when administration of therapeutic drugs typically occurs after the onset of clinical signs. Furthermore, clinical SIRS involves more complex interactions of not just LPS but other bacterial toxins with the immune system.^2^, ^3^ However, our preliminary results using a low‐dose LPS infusion model are encouraging for future clinical investigation of doramapimod in the horse, and experimental evidence in other species suggests that when administration of a p38 MAPK inhibitor is delayed after the onset of systemic inflammation, beneficial anti‐inflammatory effects and improved survival still can occur.^57^

## 5 CONCLUSIONS

Doramapimod decreased the inflammatory effects of LPS as observed by effects on heart rate, rectal temperature, blood pressure, leukocyte count, and TNF‐α and IL‐1β production. Further studies are warranted to determine the efficacy of doramapimod in clinical cases of SIRS in horses, and these preliminary results show promise for its future value as a therapeutic agent for SIRS in horses.

## CONFLICT OF INTEREST DECLARATION

This compound is the subject of a patent application filed by the University of Melbourne (Patent number PCT/AU2018/050120, filed 15 Feb 2018).

## OFF‐LABEL ANTIMICROBIAL DECLARATION

Authors declare no off‐label use of antimicrobials.

## INSTITUTIONAL ANIMAL CARE AND USE COMMITTEE (IACUC) OR OTHER APPROVAL DECLARATION

This study was approved by the University of Melbourne Animal Ethics Committee, approval number 1212538.1.

## HUMAN ETHICS APPROVAL DECLARATION

Authors declare human ethics approval was not needed for this study.
